# Molecular detection and genotyping of bovine viral diarrhea virus in Selangor, Malaysia

**DOI:** 10.5455/javar.2024.k797

**Published:** 2024-06-16

**Authors:** Nurulhidayah Khalid, Siti Suri Arshad, Nurhusien Yimer Degu, Siti Zubaidah Ramanoon, Mohammed Babatunde Sadiq

**Affiliations:** 1 Department of Veterinary Pathology and Microbiology, Faculty of Veterinary Medicine, Universiti Putra Malaysia, Serdang, Malaysia; 2 Department of Veterinary Clinical Studies, Faculty of Veterinary Medicine, Universiti Putra Malaysia, Serdang, Malaysia; 3 Department of Farm and Exotic Animal Medicine and Surgery, Faculty of Veterinary Medicine, Universiti Putra Malaysia, Serdang, Malaysia

**Keywords:** Bovine viral diarrhea, bovine viral diarrhea virus, reverse transcription polymerase chain reaction, 5’-UTR region, E2 region

## Abstract

**Objective:**

Bovine viral diarrhea (BVD) disease is a viral infection in cows caused by a single-stranded plus-sense RNA virus of the *Pestivirus* genus under the *Flaviviridae *family. The clinical manifestation of BVD mainly includes diarrhea and immunosuppression, thereby exacerbating various respiratory diseases. This study was conducted to detect and molecularly characterize the bovine viral diarrhea disease virus (BVDV) in cattle on selected farms in Selangor, Malaysia.

**Materials and Methods:**

A reverse transcription polymerase chain reaction (RT-PCR) was performed for antigen detection in 253 plasma samples collected from cows using a cross-sectional study design. We selected the 5 untranslated regions (5’-UTR) region and the E2 region to compare the genetic differences between the isolates.

**Results:**

One sample was found to be positive (1/253) following RT-PCR targeting the conserved 5’-UTR region of BVDV. Thus, BVDV antigen prevalence was 0.40% (95% confidence interval: 0.0%–2.2%). By targeting the hypervariable E2 region of the isolated virus, UPM/MAL/BVDV/D17, the virus was classified under the subgenotype BVDV-1a.

**Conclusion:**

BVDV is present and circulating on selected cattle farms in Selangor, Malaysia. Given the presence of BVDV in several subgenotypes, the screening of all incoming cattle at Malaysia’s border is pertinent to prevent the entry of other BVDV subgenotypes into the country.

## Introduction

The bovine viral diarrhea virus (BVDV) is well known as the cause of bovine viral diarrhea disease (BVD), an important disease among cattle globally [1]. The virus belongs to the *Pestivirus* genus of the *Flaviviridae* family, consisting of other members such as the Border’s disease virus (BDV) and the classical swine fever virus (CSFV). Sequence analysis of the genomic ribonucleic acids (RNAs) of these viruses and certain antigenic characterizations categorized BVDV-1 and BVDV-2 as two different species [2]. Furthermore, these four viruses differ from the genus *Flavivirus* as they encode two special proteins, specifically N^pro^ and E^rns^ while harboring the same features as the *Flaviviridae* [3]. The open reading frame carrying the genes for five structural proteins and seven non-structural proteins is arranged in the genome as follows: NH2-N^pro^, E^rns^, E1, E2, p7, NS2, NS3, NS4A, NS4B, NS5A, and NS5B-COOH [4,5]. The five structural proteins are capsid (C), three enveloped glycoproteins (E^rns^, E1, and E2), and a very small viral protein (p7) [3]. Meanwhile, the seven non-structural proteins are N^pro^, NS2, NS3, NS4A, NS4B, NS5A, and NS5B [4].

There are two biotypes of BVDV, cytopathic (CP) and non-cytopathic (NCP) [5,6]. CP BVDV causes damage in cell culture, which is characterized by vacuolation and cell lysis, whereas no deleterious effect is induced by NCP BVDV [7]. Overall, these biotypes are independent of genotypes [8]. The common detection techniques for BVDV antigen include virus isolation, antigen enzyme-linked immunosorbent assay (Ag-ELISA), reverse transcriptase-polymerase chain reaction (RT-PCR), immunohistochemistry (IHC), and nucleic acid probe hybridization [9]. Despite the gold standard for diagnosing BVDV being virus isolation, the most preferred method is RT-PCR given its cost-effectiveness, low time consumption, and high sensitivity [9]. RT-PCR can also be used to test numerous samples, such as milk, blood, saliva, follicular fluid, and tissue samples [10]. In addition, RT-PCR can facilitate the detection of both BVDV-1 and BVDV-2 by using a primer specific to the 5 untranslated regions (5’-UTR) [11].

The Ag-ELISA is simple, rapid, and useful for high-throughput applications, especially during the detection of persistently infected (PI) animals and herd screening [11]. Unlike RT-PCR, Ag-ELISA is unable to provide useful results on pooled serum samples. Meanwhile, IHC is also a common BVDV antigen detection technique but is restricted to tissue samples, particularly the ear notch [12]. The detection of BVDV antibodies is a valuable approach to assessing previous exposure to BVDV and the immune status of individual animals [9].

Despite reports from various parts of the world, there are only a few studies on BVDV in Malaysia. A previous study reported a seroprevalence of 33.2% in cattle in Selangor, and four of the five farms had at least one seropositive animal [13]. Despite the high risk of contracting the disease due to the importation of cattle from several endemic countries, Malaysia has yet to report any molecular studies on BVDV in cattle. As a result, the present study is the first molecular investigation of BVDV in cattle plasma in Selangor, Malaysia.

## Materials and Methods

### Ethical approval

Permission to conduct this research was obtained from Universiti Putra Malaysia‘s Institutional Animal Care and Use Committee (IACUC) with the following reference number: UPM/IACUC/AUP-R0303/2017.

### Study design and study location

**Figure 1.**
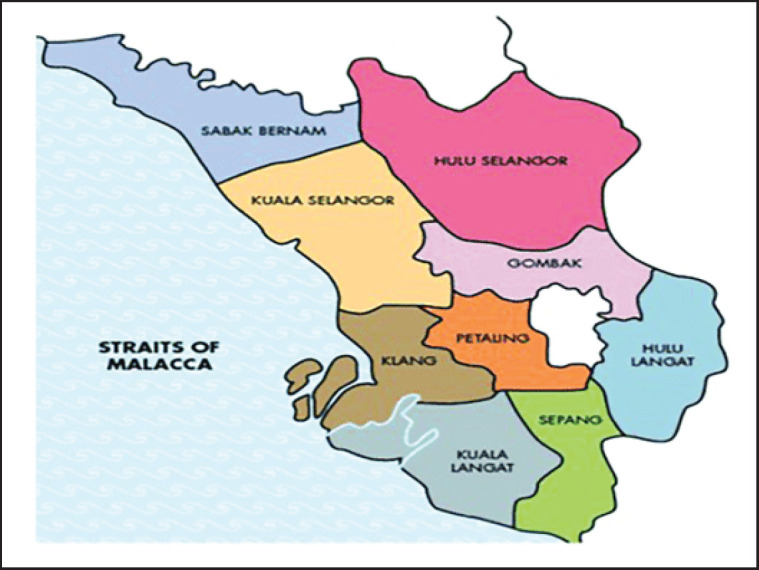
Map of Selangor and the respective districts (“Selangor state,” 2013).

This study entailed a cross-sectional design. Plasma samples were collected from clinically healthy cows from selected cattle farms in six districts of Selangor, namely Petaling, Sepang, Sabak Bernam, Klang, Kuala Langat, and Hulu Selangor (Fig. 1). The data collection was carried out from March 2016 until January 2018.

### Study size calculation

Given an expected BVDV prevalence of 21% [13], a confidence interval of 95%, and an accepted error of 5%, with a cattle population of approximately 32,463 in Selangor [14], a minimum sample size of 253 was obtained. A total of 8 cattle farms in Selangor were conveniently selected, while the animals in each farm were randomly selected. All cattle involved in this study were clinically healthy based on a physical assessment and examination of vital parameters such as respiratory rate, rectal temperature, and pulse rate. The farm identification and number of samples collected were as follows: Farm A (38 samples), B (22 samples), C (10 samples), D (14 samples), E (16 samples), F (35 samples), G (44 samples), and H (74 samples).

### Sample collection

The skin around the neck region of each cow was prepared aseptically by cleansing it with 70% ethanol. Blood was then withdrawn from the jugular vein. The blood sample was drained into an EDTA tube (BD Vacutainer, UK), held in ice, and transported to the Institutional Virology. Centrifugation of the samples was performed at 2,000x*g*, 4°C, and for 10 min (Hettich, Germany). Then, the plasma was transferred into a 1.5–ml microcentrifuge tube (Eppendorf, Germany), labeled, and kept in a −20°C freezer. The plasma samples were collected from 253 cattle on 8 farms.

### Molecular detection

Before molecular detection, a competitive ELISA assay, LSIVet™ Ruminant BVD/BD p80 kit-Serum/Milk (Thermo Fischer Scientific, USA), was conducted to categorize the samples accordingly. A total of 57 and 147 samples were categorized as positive and negative, respectively, for BVDV, whereas 52 samples were considered unknown.

We used the QIAamp Viral RNA Mini Kit (Qiagen, Germany) for the RNA extraction as described by the manufacturer. Specifically, 560 μl of prepared Buffer AVL carrier RNA-Buffer AVE was pipetted into a 1.5 ml microcentrifuge tube, followed by adding 140 μl plasma. After vortexing the mixture for 15 sec, the sample was incubated at room temperature for 10 min. Thereafter, 560 μl 100% ethanol was added to the sample, and the mixture was vortexed (Fine Vortex, South Korea) for 15 sec. The QIAamp Mini column was filled with 630 μl of the solution, ensuring that the rim was not wet. The cap was then closed before centrifugating the solution at 6,000x*g* for 60 sec. The QIAamp Mini column was placed into a 2 ml collection tube, and the filtrate in the other tube was discarded. The process was repeated until the tube was empty. Subsequently, Buffer AW1 (i.e., 500 μl) was added to the QIAamp Mini column and centrifuged at 6,000x*g* for 60 sec, which was then placed in a clean 2 ml collection tube, and the filtrate in the tube was discarded. The same procedure was performed for the buffer AW2, but the centrifugation was carried out at 20,000x*g* for 3 min. To prevent the risk of carrying over the Buffer AW2, we used a new 1.5–ml microcentrifuge tube to hold the QIAamp Mini column while discarding the filtrate in the old collection tube. Thereafter, a full-speed centrifugation was performed for 60 sec. The process of changing the microcentrifuge tube and discarding the filtrate was repeated. The QIAamp Mini column’s cap was opened carefully before adding 60 μl of Buffer AVE at room temperature. The process was completed by closing the cap before incubating and centrifugating at 6,000x*g* for 1 min. All the RNA extracts were stored at −70°C until assayed.

The viral RNA extracted was subjected to one-step RT-PCR (Promega, USA) using both 5’ untranslated region (5’-UTR) and envelope 2 (E2) primer sets. Two sets of primers were used in this study to amplify two regions of BVDV. Primer 324/326 was obtained from the previously published work that flanked the 5’-UTR with a product size of 288 bp [15]. Meanwhile, primer E2F/E2R was designed from the reference strain BVDV1 (NADL strain) using Primer-BLAST (https://www.ncbi.nlm.nih.gov/tools/primer-blast/) to amplify the envelope 2 (E2) regions with an expected band product of 700 bp (Table 1).

BVDV-1 (ATCC^®;^ VR-534™) is the bovine viral diarrhea virus type 1 (BVDV-NADL strain) reference strain used in this study. The reference strain was propagated in bovine turbinate cells (ATCC, USA) and used as a positive control. The Access RT-PCR System (Promega, USA) was employed for the RNA transcription and amplification. Briefly, the RT-PCR master mixture was prepared in a volume of 25 µl. The mixture comprised 2 µl of 25 mM magnesium sulfate (MgSO_4_), 5 µl of 5X Flexo buffer, 0.5 µl each of RNase inhibitor, dNTP, forward and reverse primers (324/326 and E2F/E2R), reverse transcriptase, Taq polymerase, and 10 µl of reference BVDV RNA template, and 4.5 µl of RNase-free water. Pulse-vortexing was performed to mix the PCR mixture thoroughly and spun down (VWR^®;^ Mini Centrifuge, USA). The thermal cycling protocol for amplification of the 5’-UTR gene of BVDV was set as follows: one cycle of initial heating temperature at 45°C for 45 min, an initial denaturation temperature at 94°C for 2 min, another 45 cycles of denaturation at 94°C for 30 sec, 56°C annealing temperature for 1 min for primers 324/326, and 68°C of extension for 2 min. The setting for the final extension temperature was 68°C for 10 min (Eppendorf, Germany).

The thermal cycling protocol for amplification of the E2 gene of BVDV was set as follows: one cycle of initial heating temperature at 45°C for 45 min, an initial denaturation temperature at 94°C for 2 min, another denaturation at 94°C for 45 cycles and 30 sec, 50°C annealing temperature for 1 min for primers E2F/E2R, and extension at 68°C for 2 min. The final extension temperature was set at 68°C for 10 min (Eppendorf, Germany). After the PCR reaction, electrophoresis was performed using 2.0% (w/v) agarose gel (2.0 g agarose (SeaKem, USA) in 100 ml 1X TAE (40 mM Tris-acetate, 0.5M EDTA, pH 8.2) and a buffer (FirstBase, Malaysia) added with 2.0 µl RedSafe (iNtRON Biotechnology, South Korea) to detect the nucleic acid products. Thereafter, 5 µl of PCR product was mixed with 1 µl of 6X Blue Green Loading Dye^®;^ (Promega, USA). Meanwhile, 100 bp and 1 kb markers (Promega, USA) were used in the electrophoresis of 5’-UTR and E2 PCR products, respectively, and were loaded into the casted gel. Electrophoresis was run at 80 V, 400 mA for 25 min (BioRAD, USA). The stained agarose gel was placed onto a UV transilluminator (Bio-Red, USA), visualized, and photographed in gel documentation systems.

**Table 1.** Primers were used for amplification of the 5’-untranslated region (5’-UTR) and envelope 2 (E2) of BVDV by One Step RT-PCR.

Primer	Targeted region	Primer sequences (5'–3')	Size (bp)	Reference
324	5'-UTR	TAGCCATGCCCTTAGTAGGAC	288	[15]
326	5'-UTR	ACTCCATGTGCCATGTACAGC	288	[15]
E2F	E2	ACTTTAAATTTG GACTYTGCC	700	In this study
E2R	E2	TCCAGGTCAAACCARTATTG	700	In this study

### Genotyping

Several regions of the BVDV genome have been employed in phylogenetic studies of BVDV to compare the genetic variation between isolates. We selected the 5’-UTR region before proceeding to the E2 region. A phylogenetic analysis was performed using the 5’-UTR coding sequences of 22 BVDV genotypes (5 BVDV-1a, 4 BVDV-1b, 2 BVDV-1c, 2 BVDV-1d, 2 BVDV-2a. 4 BVDV-2b, 1 BVDV2c, 2 BVDV-3), 1 pronghorn virus, and 2 CSFVs to determine the genetic relationships (Table 2). This was followed by using the E2 coding sequences of 12 BVDV genotypes (4 BVDV-1a, 2 BVDV-1b, 2 BVD-1c, 1 BVDV-1d, and 2 BVDV-2. 1 BVDV-3) and 1 CSFVs to examine the genetic relationships (Table 3). Both phylogenetic analyses were executed using the MEGA 7.0 software, specifically the neighbor-joining method. Meanwhile, the evolutionary distances were computed via the Tamura 3-parameter model with 1,000 bootstrap replicates in the phylogenetic tree. Multiple sequence alignments were conducted using the ClustalW program.

**Table 2.** *Pestivirus* strains used in the phylogenetic studies of 5’-UTR partial sequences of bovine viral diarrhea virus (BVDV).

No.	***Pestivirus* strain**	Genotype	GenBank accession no.
1	1	1a	EF210347
2	NADL	1a	AJ133739
3	9	1a	EF210355
4	MSGOCOA20	1a	MF347398
5	MSGLCOA260	1a	MF347400
6	MRI5087	1b	LT903549
7	MRI0051	1b	LT900588
8	Osloss	1b	M96687
9	Hercules Canada	1b	JX297517
10	Mogilla	1c	JQ43605
11	Grafton	1c	JQ43607
12	MRI2347	1d	LT902505
13	MRI1690	1d	LT902023
14	MSGOCOA221	2a	MF491395
15	MSGOCAA217	2a	MF491394
16	MSGOCAB233	2b	MF491397
17	MSGLCAB226	2b	MF491396
18	SD1301	2b	KJ000672
19	Hokudai Lab 09	2b	AB567658
20	Short	2c	MH231149
21	Th04 KhonKaen	3	NC012812
22	HoBi	3	AB871953
23	Pronghorn virus	Pronghorn	NC024018
24	JSZL	CSFV1	KT119352
25	GXF29	CSFV2	KP233070

## Results

### Descriptive findings

Following the RT-PCR assay, only one farm (Farm D) out of the 8 farms was positive for BVDV antigen. Only one sample was positive out of the 14 animals tested on this farm. Meanwhile, no BVDV antigen was detected in the other 7 farms. The prevalence of BVDV antigen in Farm D was 7.1% (95% CI: 2.0–33.9), while the overall prevalence in all the sampled cattle in Selangor was 0.40% (95% CI: 0.0–2.2) (Table 4). Statistically, BVDV prevalence was not significantly different between farms (*p* = 0.556, χ^2^ = 17.139).

### RT-PCR, BLAST analysis, and construction of phylogenetic tree

**Figure 2.**
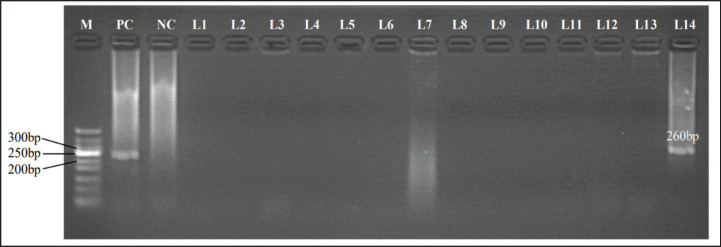
Representative electrophoresis of RT-PCR assay of fourteen plasma samples of cattle from farm D using primer set 324/326 with an annealing temperature of 56°C to yield a product of approximately 260 bp of 5’-UTR region of BVDV. Lanes L1, L3 to L5, L10, and L14 were plasma samples from seronegative cattle. Lanes L2, L6, and L7 were plasma samples from unknown BVDV antibody status cattle. Lanes L8, L9, and L11 to L13 were plasma samples from seropositive cattle. Lane M: 100 bp DNA ladder (Promega, USA), Lane PC: positive control, Lane NC: negative control, Lanes L1 to L13 showed negative amplifications, Lane L14 showed positive amplification from a cattle ID: D17.

Following electrophoresis, primer sets 324/326 yielded the product of approximately 260 bp of the 5’-UTR region, and primer sets E2F/E2R gave the product of approximately 700 bp of the E2 region of BVDV. The sample designated as D17 (lane 14) was obtained from a seronegative BVDV animal of farm D and was positive for the 5’-UTR conserved region (Fig. 1) and the E2 hypervariable region of BVDV (Fig. 2).

A phylogenetic tree was built from nucleotide sequences of a conserved region of 5’-UTR of the UPM/MAL/BVDV/D17 positive sample of cattle. The sequences were compared with related sequences obtained from GenBank. Blast analysis with the genome sequences revealed that the sequenced isolate of UPM/MAL/BVDV/D17 5’-UTR region shared a high nucleotide identity ranging from 91.57% to 95.20% with other BVDV1 strains. Table 2 depicts all the reference viruses included in the analysis, as well as their corresponding GenBank accession numbers. The accession number for the local isolates UPM/MAL/BVDV/D17 was MW147360.

**Table 3.** *Pestivirus* strains used in the phylogenetic studies of E2 partial sequences of bovine viral diarrhea virus (BVDV).

No.	*Pestivirus* strain	Genotype	GenBank accession
1	V077	1a	KX170168
2	V049	1a	KX170167
3	TGAC-B2 strain	1a	Z54185
4	CHL193	1a	JF776635
5	GS31	1b	KF048853
6	95409	1b	AFX69721
7	JINAN01	1c	KF048843
8	GS35	1c	KF048842
9	ISO122	1d	KF048844
10	BVDV2-Parker	2	AF145971
11	BVDV2-AzSpln	2	AF145970
12	Italy-28011-A	3	AEZ57100
13	CSFV-UP-GZ-NG39-10	CSFV	KC533787

**Table 4.** Prevalence of bovine viral diarrhea virus (BVDV) in cattle in Selangor as detected by RT-PCR assay.

No.	Farm	No. of samples	RT-PCR results		Prevalence (%)	95% CI
			+ve Ag	-ve Ag		
1	A	38	0	38	0	0.0–9.3
2	B*	22	0	22	0	0.0–15.4
3	C	10	0	10	0	0.0–30.8
4	D	14	1**	13	7.1	2.0–33.9
5	E	16	0	16	0	0.0–20.6
6	F*	35	0	35	0	0.0–10.0
7	G	44	0	44	0	0.0–8.0
8	H	74	0	74	0	0.0–4.9
Overall		253	1	252	0.40	0.0–2.2

* Seronegative farm;

** seronegative animal.

**Figure 3.**
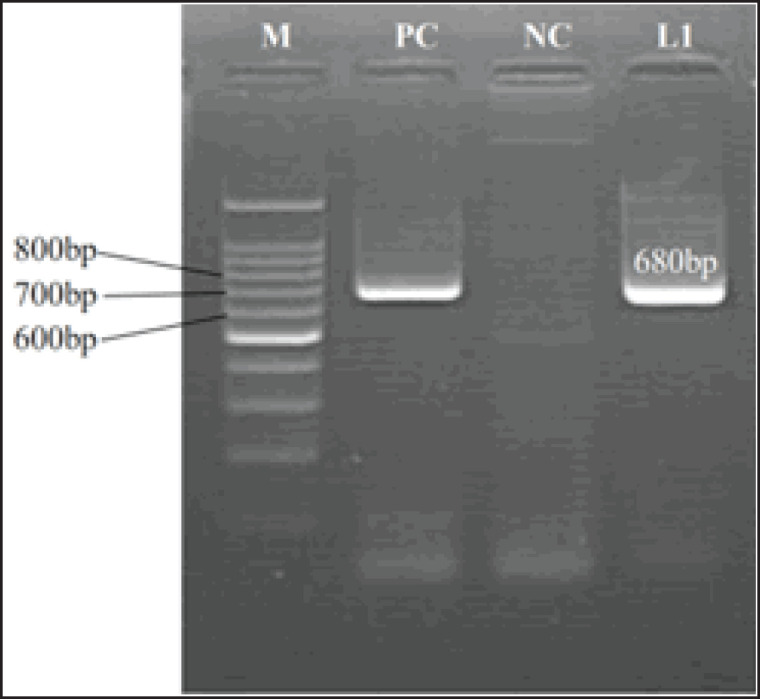
Electrophoresis of RT-PCR assay of a plasma sample of cattle from farm D (cattle ID: D17) using primer pair E2F/E2R with an annealing temperature of 50°C to yield the product of approximately 680 bp of E2 hypervariable region of BVDV. Lane M: 1kB bp DNA ladder (Promega, USA), Lane PC: positive control, Lane: NC negative control, Lane L1 depicts positive amplification.

Based on the neighbor-joining method and the ClustalW alignment used for the phylogenetic analysis, the isolate: UPM/MAL/BVDV/D17 was closely related to the BVDV1a subgenotype, which includes the isolate MSGLCOA260, isolate MSGOCOA20, isolate 9, isolate 1, NADL strain, and that this isolate clustered distinctly from other BVDV1 subgenotypes (Fig. 3).

**Figure 4.**
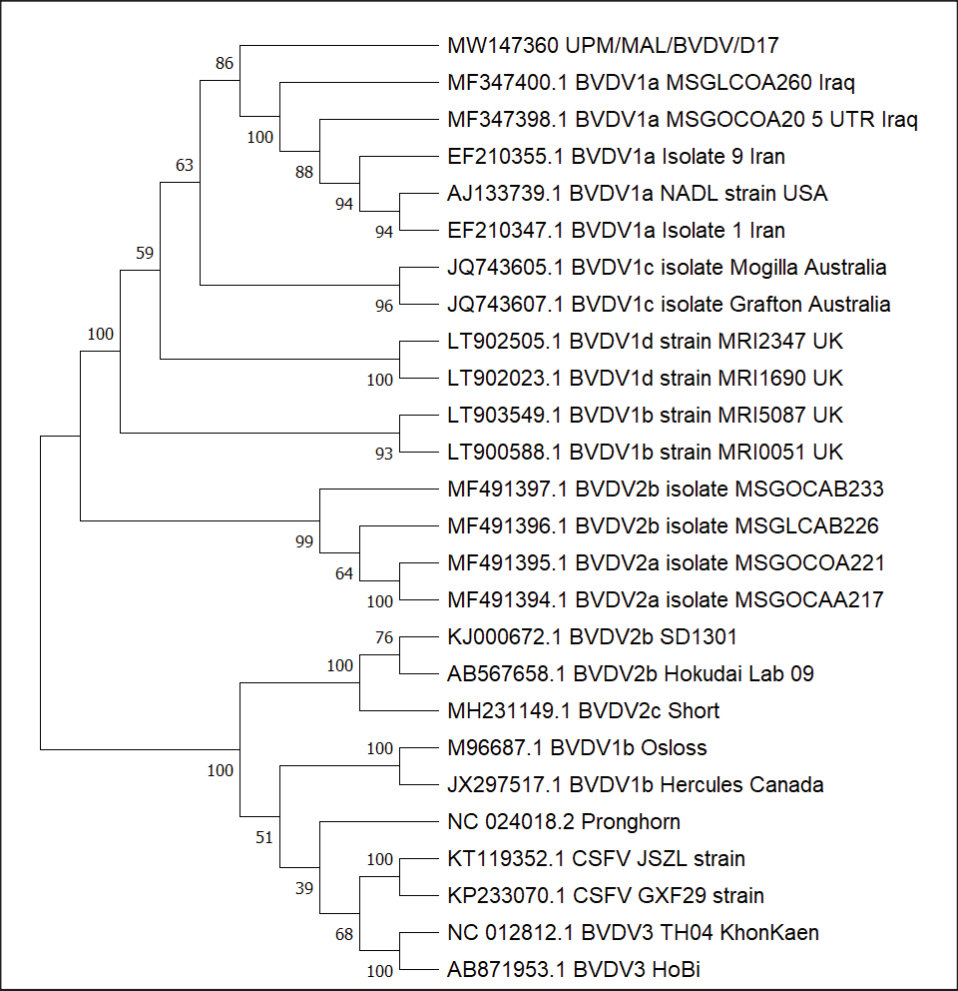
Phylogenetic analyses based on the conserved region of 5’-UTR coding sequences of BVDV/MAL/D17 with other 22 BVDV genotypes (type 1, type 2, and type 3) and 2 CSFV and 1 pronghorn virus were conducted to examine the genetic relationships. The CSFV JSZL strain and CSFV GXF29 strain were the outgroups. The numbers in branches indicate bootstrap support of 100 replications. The local isolate BVDV/MAL/D17 (red box) is clustered within the BVDV subgenotype 1a and was closely related to other strains and isolates from Iraq (MSGLCOA260 and MSGLCOA20), Iran (Isolate 9 and Isolate 1), and USA (NADL strain).

Blast analysis with the genome sequences revealed that the isolate UPM/MAL/BVDV/D17 E2 region shared a high nucleotide identity ranging between 84.79% and 99.69% with other BVDV1 strains. The accession number of the local isolates UPM/MAL/BVDV/D17 is MW147359. Upon analyzing the E2 coding sequences of the isolate, the UPM/BVDV/MAL/D17 was closely related to the BVDV1a subgenotype, which includes the isolate CHL 193, TGAC-B2 strain, isolate V077, and isolate V049 (Fig. 4).

## Discussion

The standard diagnostic method for BVDV is the RT-PCR assay, which is less time-consuming and less laborious as compared to isolation in cell culture [11]. In addition, acutely infected, PI, and vaccinated animals can be examined for BVDV antigen by using the RT-PCR assay. Therefore, a single positive RT-PCR is not sufficient to ascertain an animal’s clinical status.

The BVDV RT-PCR positive animal in this study was a 10-year-old breeding bull of a seropositive Farm D. The bull was tethered in a group of other breeding cows and occasionally left to graze in a plot near the farm with the same group. The competitive ELISA assay and farm record indicated that the bull was seronegative, but the breeding cows of the same group sampled were seropositive. Since the bull was not used for breeding, as the farmer in Farm D preferred AI, there is a possibility that the cows were infected through direct contact rather than natural breeding. The infection status of the bull could not be determined in the present study as the test was conducted once, while PI animals could be identified when they are antigen-positive for more than two tests in 3–4-week intervals.

There are several possibilities regarding the clinical status of the BVDV-positive RT-PCR bull. In acute infections, the viral antigen is found mostly in the body fluids between 5 and 9 days post-infection [9]. In an experimental study by Kelling et al. [16], the viral antigen was found particularly in the lymphoid tissues but was absent in the bone marrow of acutely infected calves. Acute BVDV infection can also affect reproductive performance. Chronic infection in the testes and persistent virus shedding may occur in the semen for 4–5 months after infection. A previous study also reported the detection of BVDV in the cell lining of the seminiferous tubules [17]. Overall, the viraemic state of animals with acute infection lasts for a shorter period, ranging from 2–3 weeks before seroconversion [18]. The virus can also be eliminated from most tissues in less than 2 weeks post-infection, and these acutely infected animals do not transmit the virus to other herdmates [19]. Therefore, samples from bulls with acute BVDV infection were collected 5–9 days post-infection, when viremia occurred and before seroconversion. Moreover, paired acute and convalescent sera collected 3 weeks after the acute stage may incriminate or exclude BVDV infection by serological testing.

Another possibility concerning the BVDV status of the cattle is persistent infection. This condition occurs because of NCP BVDV infection in early gestation, in which the fetus develops immune tolerance and fails to develop antibodies against the virus due to the immature and naïve immune system. Nevertheless, they later developed a life-long immune tolerance against the pathogen. Thus, PI animals are usually seronegative. The present findings revealed that the RT-PCR-positive bull was seronegative. However, PI animals can only be identified if they are positive for BVDV antigen screening tests twice or more. Sampling can be repeated in 3 to 4-week intervals. Various tests are available for PI detection, such as the antigen-capture ELISA, IHC, and RT-PCR, which all offer high sensitivity and specificity when tested individually. Nonetheless, antigen-capture ELISA requires a fresh ear notch but appears to be the most robust test for BVDV detection. Upon detecting BVDV on a farm, multiple tests are recommended to ensure the disease is effectively controlled. It is necessary to test all young animals on the farm, given that a PI seropositive calf reflects the need to test the dam as well. Consequently, to prevent the infection from spreading further, all PI animals are to be culled immediately from the farm.

Following the identification of PI animals, other animals on the farm are usually administered a modified CP BVDV live vaccine virus. There is a risk of the occurrence of MD due to these combined infections (NCP BVDV and CP BVDV). Modified BVDV live virus vaccines may preserve some virulence, thereby depleting the lymphoid tissues, particularly the peyer patches. The functions of the neutrophils and lymphocytes could also be altered post-vaccination [18]. The tendency for vaccines to be contaminated by virulent BVDV cannot be ruled out, which could lead to calves with persistent BVDV infection upon vaccination of pregnant animals. Bovine serum is used as a source of protein obtained from BVDV-infected animals when preparing vaccines using cell culture [20]. Extra-label administration of vaccines to pregnant animals may also lead to virus shedding and finding their way into PI calves, especially in conducive circumstances [21]. In Malaysia, BVDV vaccines are not used to control the disease. However, the BVDV RT-PCR-positive bull was imported from Australia, where BVDV vaccines are widely used [22]. Furthermore, many producers prefer to use modified live vaccines, as the protection provided by the vaccine is broader and longer than that of the killed vaccines.

**Figure 5.**
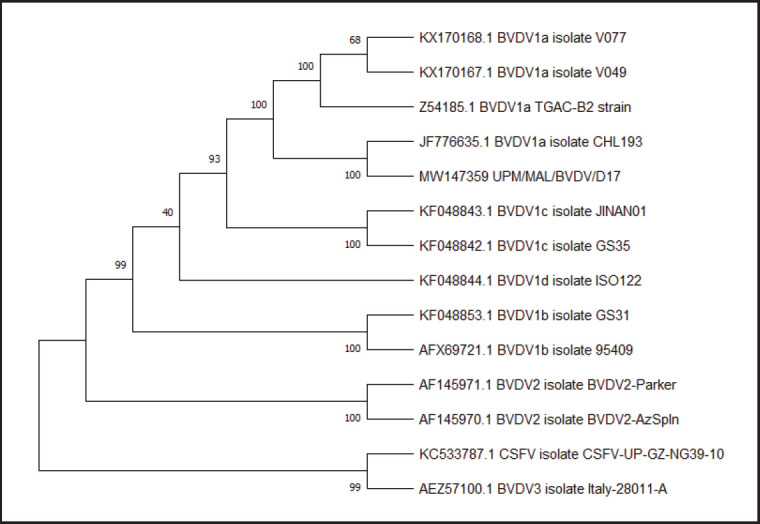
Phylogenetic analysis based on the hypervariable region of E2 coding sequences of BVDV/MAL/D17 with 12 other isolate/strain BVDV genotypes (type 1, type 2, and type 3) and 1 CSFV was conducted to examine the genetic relationships. CSFV was the outgroup. The numbers in the branches indicate bootstrap support of 100 replications. The local isolate BVDV/MAL/D17 (red box) is clustered within BVDV subgenotype 1a and was closely related to other strains and isolates from Chile (isolate CHL 193), Germany (TGAC-B2 strain), and Canada (isolate V077 and V049).

The phylogenetic trees represent the evolutionary relationships between the BVDV isolates in this study and their counterparts (Fig. 5). Furthermore, the branching pattern demonstrates how BVDV isolates in Malaysia and others evolved from a set of similar ancestors. The local 5’-UTR UPM/MAL/BVDV/D17 subgenotypes were identified as BVDV1a subgenotypes. The local IBDV sequence reflected the closest relative with 2 isolates from Iraq, namely MSGLCOA260 BVDV1a isolate, MSGOCOA20 isolate [23], 2 isolates from Iran, namely isolate 9 and isolate 1 Iran [24], and the NADL strain of the USA [25]. Hence, the local UPM/MAL/BVDV/D17 isolate is clustered within BVDV subgenotype 1a and is closely related to other strains and isolates from Iraq, Iran, and the United States.

The genomic sequence analysis of E2 UPM/MAL/BVDV/D17 was further identified as the BVDV subgenotype 1a. The local IBDV sequence was closely related to isolate CHL 193 from Chile [26], isolate V077, and isolate V049 from Canada [27]. Hence, the local isolate is clustered within the BVDV subgenotype 1a and is closely related to other strains and isolates from Chile, Germany, and Canada.

It is difficult to explain the genetic revolution of the local BVDV1a isolates with that of BVDV1a from Iran, Iraq, the USA, Chile, Germany, and Canada, as these countries are not located nearby. In addition, there is no documented record indicating the importation of cattle from these countries into Malaysia. However, Saidi et al. [28] reported the detection of BVDV2 in local pigs by the Department of Veterinary Services, Malaysia. Notably, the report was the first detection of BVDV in a swine population in Sabah, East Malaysia. The source of the infection was not elucidated, but the use of imported live animals is a common practice for breeding purposes in the local swine industry. The imported cattle in Malaysia usually came from BVDV-endemic countries such as Australia and Thailand. The majority of BVDV genotypes found in cattle in Australia were BVDV-1c, followed by subgenotypes 1a, 2a, and 1b [20]. In Thailand, a third new genotype of BVDV-3, which was previously known as the HoBi-like strain, was first detected in a calf in 2006 [29]. Genotype BVDV1a is prevalent in cattle in Central and East Java, Indonesia, together with genotypes 1b and 1c [7]. Sharing a land border in Kalimantan between Malaysia and Indonesia could permit the uncontrolled movement of animals between the two countries. Additionally, Bali cattle were a subject of interest to the Malaysian government a few years ago, when the cattle were being imported for promotion and breeding purposes.

There are important limitations in this study, particularly in terms of sampling location, limited information from the sampled farms, and the fact that only one animal was positive for the BVDV antigen. All the sampled farms were located in one state in Malaysia, which reflects the low generalizability of the results. Detailed information on previous exposure to BVDV and vaccination history was not available from each farm. In addition, extensive discussion on the local UPM/MAL/BVDV/D17 isolate could not be performed since only one animal was positive for the BVDV antigen. Nevertheless, the finding highlights the need for further characterization of the variation and geographical distribution of BVDV in Malaysia.

## Conclusion

This research demonstrated the presence of BVDV antigen in cattle farms in Malaysia. Although only one of the sampled cattle was positive for the BVDV antigen, the animal may have infected other cattle on the farm. Genotyping of UPM/MAL/BVDV/D17 5’-UTR and E2 regions demonstrated that the local isolate belonged to the subgenotype BVDV1a, which is one of the several subgenotypes present in Indonesia. Nevertheless, BVDV is also prevalent in countries like Thailand, but in other subgenotypes (BVDV3). Moreover, countries exporting live cattle to Malaysia do have BVDV1a but of other different subgenotypes. For instance, BVDV1c is the most prevalent in Australia. Given the significant economic loss associated with BVD, the Malaysian government must implement effective measures to prevent the introduction of other BVDV variants to the country’s cattle and other ruminants. A screening program is crucial to ensure cattle are free from the pathogen before importation into Malaysia. The current findings also highlight vital information for further characterization of the variation and geographical distribution of BVDV in Malaysia.

## List of Abbreviations

5’-UTR, 5 untranslated regions; BVD, Bovine viral diarhoea; BVDV, Bovine viral diarhoea virus; CSFV, Classical swine fever virus; CP, Cytopathic; NCP, Non-cytopathic; PI, Persistently infected; RT-PCR, Real time polymerase chain reaction.
